# Aidi injection as adjunctive treatment to gemcitabine-based chemotherapy for advanced non-small cell lung cancer: a systematic review and meta-analysis

**DOI:** 10.1080/13880209.2021.1973038

**Published:** 2021-09-19

**Authors:** Sitong Guo, Yan Li, Henghai Su, Mingyu Meng, Jiaxi Xi, Guangyan Mo, Xiaoyu Chen

**Affiliations:** Department of Pharmacy, The People's Hospital of Guangxi Zhuang Autonomous Region, Nanning, Guangxi, China

**Keywords:** Traditional Chinese medicine, advanced NSCLC, GBC

## Abstract

**Context:**

Aidi injection is one of the most commonly use antitumor Chinese medicine injections for advanced non-small cell lung cancer (NSCLC). It is made from the extraction of *Astragalus, Eleutherococcus senticosus, Ginseng*, and *Cantharis.*

**Objective:**

To evaluate the efficacy and safety of Aidi injection in combination with gemcitabine-based chemotherapy (GBC) for advanced NSCLC.

**Materials and methods:**

PubMed, Embase, Cochrane Library, Chinese Biological Medicine, China National Knowledge Infrastructure, Wanfang, and VIP were searched for relevant randomised controlled trials (RCTs) comparing Aidi injection plus GBC treatment with GBC alone in NSCLC, from inception up to October 2020. The primary outcomes were objective response rate (ORR), and disease control rate (DCR). Secondary outcomes were quality of life (QOL) and adverse drug reactions (ADRs). The quality of evidence was rated using the GRADE approach. This study was registered with PROSPERO: CRD42021221225.

**Results:**

In total, 54 RCTs involving 4318 NSCLC patients were included in this meta-analysis. Compared with GBC alone, Aidi injection plus GBC significantly improve ORR (risk ratios [RR] = 1.38, 95% confidence interval [CI] 1.29–1.48), DCR (RR = 1.15, 95% CI 1.12–1.19), QOL (RR = 1.71, 95% CI 1.54–1.89), and reduced the risk of gastrointestinal toxicity, thrombocytopenia, neutropenia, liver injury, renal injury, and anaemia. The evaluation results of the evidence ranged from moderate to low.

**Conclusions:**

Current moderate evidence revealed that Aidi injection as an adjunctive treatment to GBC was associated with superior benefits in patients with advanced NSCLC and alleviate toxicities. High-quality RCTs are needed to further confirm the results.

## Introduction

Globally, cancer is a chief cause of death in malignant tumours, and ∼85% of cancers are diagnosed as non-small cell lung cancer (NSCLC) (Siegel et al. 2020). Moreover, approximately two-thirds of NSCLC cases have already progressed to stage III/IV, at the time of diagnosis (Morgensztern et al. 2010; Bray et al. 2018; Siegel et al. 2020). In NSCLC patients with advanced disease progression and metastasis, platinum-based chemotherapy (PBC) is still the recommended treatment, which is constituted with platinum compounds and third-generation chemotherapy agents, such as TC/TP/TO, GC/GO, or NP/NO (Baggstrom et al. 2007; Lang et al. 2009; de Castro et al. 2017). However, the adverse drug reactions (ADRs) associated with PBC treatment seriously reduce the quality of life (QOL) in patients with NSCLC. Therefore, a better and more effective treatment development is urgently needed, especially for NSCLC patients.

From thousands of years, traditional Chinese medicine (TCM) has practiced, and alternative or combined treatment is mainly used in TCM for cancers (Efferth et al. 2007; Lin et al. 2019; Wu et al. 2019; Xiang et al. 2019). Aidi injection is a new multi-targeted antitumor Chinese medicine injection that is used as an adjuvant therapy for NSCLC. Aidi is composed of many kinds of Chinese medicines and is refined using modern preparation technology. Its main ingredients include *Ginseng, Astragalus, Cantharide,* and *Acanthopanax*. Various clinical studies have shown that Aidi injection inhibits growth, promotes apoptosis, and regulates the immune function in tumour cells (Shi et al. 2001).

Gemcitabine is an essential chemotherapeutic drug. Gemcitabine-based chemotherapy (GBC) as an important chemotherapy regimen in NSCLC, is composed of gemcitabine plus cisplatin or oxaliplatin (GP or GO). At present, there are many reports on Aidi injection combined with the GP regimen used in NSCLC treatment (Huang et al. 2017; Zhao and Li 2019). Although many clinical studies have evaluated the adjuvant effect of Aidi injection in chemotherapy, each study had a different sample size, and the results varied among studies; therefore, there is still a lack of relevant systematic evaluation. This study aimed to systematically evaluate the efficacy and safety of Aidi injection as an adjunctive treatment to GBC for NSCLC from the perspective of evidence-based medicine to provide evidence for clinical medication.

## Materials and methods

This meta-analysis was performed by following the Preferred Reporting Items for Systematic Reviews and Meta-Analyses guidelines (Moher et al. 2009). This study has been registered with the International Prospective Register of Systematic Reviews (PROSPERO): CRD42021221225.

### Searching strategies

Two authors (Sitong Guo and Yan Li) used MeSH and free word to implement all the retrievals and completed the search strategy. The study search was carried out in PubMed, Embase, the Cochrane Library, the Chinese Biological Medicine Database, the China National Knowledge Infrastructure database, the Wanfang Database, and the Chinese Scientific Journal Database (VIP) from inception to October 2020. For Pubmed databases, the following search strategy were used: ("Lung Neoplasms"[Mesh] OR "Carcinoma, Non-Small-Cell Lung"[Mesh] OR nsclc[Title/Abstract] OR lung cancer*[Title/Abstract] OR lung carcinoma*[Title/Abstract] OR lung neoplasm*[Title/Abstract] OR lung tumour*[Title/Abstract] OR lung tumour*[Title/Abstract] OR non-small cell*[Title/Abstract] OR nonsmall cell*[Title/Abstract]) AND ("Aidi" [Supplementary Concept] OR Addie[Title/Abstract] OR Aidi[Title/Abstract]), with sensitivity- and precision-maximizing version search filters. In addition, all the searched articles were reviewed to identify potential randomised controlled trials (RCTs), and the language or publication status was not restricted.

### Inclusion and exclusion criteria

They include studies that met the following criteria: (1) Patients who participated in the study were diagnosed with stage III-IV NSCLC. Furthermore, the histopathological and cytological diagnostic criteria and TNM staging system were the diagnostic basis, and age, sex, or race was not limited (Mountain 1989). (2) RCTs evaluating the efficacy and safety of Aidi injection as adjuvant treatment for stage III-IV NSCLC, regardless of whether blinding methods were mentioned. (3) All patients were administered a basic chemotherapy regimen of gemcitabine combined with carboplatin, cisplatin, or oxaliplatin. Based on this, one of the included Aidi injections was used for the patients in the experimental group, and those in the control group were administered only the chemotherapy regimens of gemcitabine combined with carboplatin, cisplatin, or oxaliplatin. Furthermore, therapy should have been administered to the patients who had suffered complications during the therapeutic process. (4) Outcomes included disease control rate (DCR), objective response rate (ORR), quality of life (QOL), and adverse drug reactions (ADRs).

Exclusion criteria were: (1) No RCT. (2) A negative diagnosis of NSCLC. (3) Any differences in the intervention except for Aidi injection treatment between the two groups. (4) Patient baseline data inconsistent. (5) Unavailable full-text article or unextractable data.

### Selection of studies

Two authors (Sitong Guo and Yan Li) strictly selected the studies according to the predefined inclusion criteria. Any dissension about selection was resolved through discussion with the third author (Xiaoyu Chen).

### The assessment of methodological bias risk

The Cochrane evaluation handbook (version 5.4.0) was used to evaluate the methodological bias risk in all studies (Higgins et al. 2020). The bias risk indices were the random sequence generation, allocation concealment, blinding of participants, personnel, and outcome assessors, lost to follow-up, selective reporting, and other biases (baseline incomparable). Assessing the risk of bias, all studies were divided into three levels (“Yes”, “No”, or “Unclear”), by the two reviewers independently. Any disagreements of judgement about “high, low or unclear risk” were resolved by discussion between themselves or with a third reviewer (Sitong Guo).

### Definition of outcome measures

ORR and DCR were the mainly indices of tumour responses. Generally, the World Health Organisation (WHO) guidelines or Response Evaluation Criteria in Solid Tumours (RECIST) were used as the assessment criteria for tumour responses (Miller et al. 1981; Watanabe et al. 2003). Complete response (CR): At least 4 weeks later, all known tumours disappeared. Partial response (PR): At least 4 weeks later, more than 50% of the tumour lesions decreased. No change (NC): A 50% decrease in total tumour size was not achieved, or it had no 25% increase in the size of one or more measurable lesions. Progressive disease (PD): There was 25% or more increase in the size of one or more measurable lesions or the appearance of new lesions. ORR be defined as CR plus PR, and DCR be defined as CR plus PR plus NC. According to the Karnofsky Performance Status Scale (KPS), the improvement of QOL was considered if the KPS evaluation score was increased by more than 10 points after treatment (Yates et al. 1980; Li et al. 2006). According to the WHO standards or common terminology criteria for adverse events version (CTCAE), ADRs were established as gastrointestinal toxicity, liver or renal injury, thrombocytopenia, anaemia, neutropenia, etc. (Miller et al. 1981; Trotti et al. 2003).

### Data extraction

All data were extracted and recorded in a consolidation data form by two reviewers (Guo and Li). The following were the data that needed to be collected: (1) basic information of the study: first author and publication date; (2) features of the participants: number of participants in the experimental group and control group, proportion of males/females, average age and specific intervention; and (3) outcome data that included ORR, DCR, QOL, and ADRs.

### Risk of bias in individual trials

According to the Cochrane Handbook, Sitong Guo, and Yan Li appraised the risk of bias for all included trials (Higgins et al. 2020). Random sequence generation (selection bias), allocation concealment (selection bias), blinding of participants and personnel (performance bias), blinding of outcomes assessments (detection bias), incomplete outcome data (attrition bias), selective reporting (reporting bias), and other biases were evaluated. In case of any dispute, the third reviewer (Henghai Su) participate in the discussion until all included trials were classified as three levels (“Yes”, “No”, or “Unclear”).

### Statistical analysis

The Cochrane Collaboration recommends using Review Manager (RevMan) 5.3 and STATA 14.0 (Stata Corporation, College Station) to perform this meta-analysis. Risk ratios (RR) and 95% confidence intervals (CI) were used to express all data, and statistical significance was set at *p* < 0.05. On account of the potential heterogeneity between the trials, the present study employed the *χ*^2^-based *Q*-test to examine the statistical test for heterogeneity and the *I^2^* index described the heterogeneity among the trials. The selection of the mathematical statistics method, which was adopted to calculate the RR and the 95% CI, depended on the results of *p* and *I^2^*: (1) the random-effects model (*p* ≤ 0.1, *I^2^* > 50%); (2) the fixed-effects model (*p* ≥ 0.1, *I^2^* ≤ 50%). If the number of involved studies reached ten or more, the potential publication bias had to be explored using funnel plots. In order to prove the clinical heterogeneity and its influence on ORR and DCR the usages (days and doses) of Aidi injection were the basis for subgroup analysis. Sensitivity analyses and trial sequential analysis (TSA) were also conducted by excluding each study to investigate the confidence of the outcomes; statistical heterogeneity does not exist if the combined estimate of sensitivity analyses in 95% CI; the result is reliable and the sample size is sufficient when the *Z*-score curve (blue line) crossed the statistical significance boundary (red polylines) and the intersection is above the dark green line. Meta-regression analysis (MRA) was used to assess the potential heterogeneity and confounding variables; the result is not robust when *p* ≤ 0.01.

### Quality of evidence

The GRADE approach was adopted to assess the overall quality of the evidence for each outcome by two reviewers (Guo and Li) independently (Guyatt et al. 2008). In case of any dispute, the third reviewer would participate in the discussion, until all included trials were classified into four levels (“high”, “moderate”, “low”, or “very low”). There are five domains could that could downgrade the quality of evidence: (1) limitation of the study design (one level should rate down if the evidence was considered to have a high risk of bias, poor robustness or unclear methodological bias risk in most cases), (2) Inconsistency (the statistical heterogeneity and result of poor robustness should be taken into consideration seriously), (3) Indirectness (the participants, intervention, outcomes, or comparison of the study did not match with the objectives of this study), (4) Imprecision (the total sample size did not reach the optimal information size, and the sample size for each indicator was less than 300 cases), (5) Publication bias (asymmetry of funnel plot was present and the efficacy was over-estimated or ADRs underestimated).

## Results

### Search results

**Figure 1.**
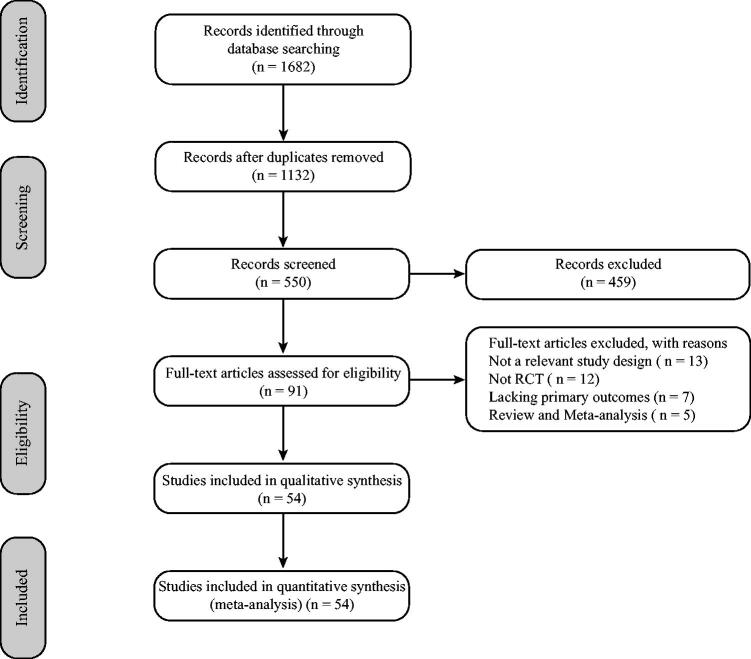
Flow diagram of study selection.

The database identified 1682 literature citations, including 1132 duplicates, 459 records (reviews, case reports, and animal experiments), and 91 records (further assessment for eligibility). Then, after reading the remaining 91 full texts carefully, further records were excluded because of the following four reasons: (1) study design was not relevant (*n* = 13); (2) not an RCT (*n* = 12); (3) lack of primary outcomes (*n* = 7); and (4) review and meta-analysis (*n* = 6). Finally, 54 RCTs were included in this study (Figure 1).

### Characteristics of the included trials

Fifty-four RCTs involving 4318 stage III-IV NSCLC participants were included in this meta-analysis. Among the 4318 participants, 2504 were men and 1473 women aged 21–80 years. The patients in the experimental groups (2118 cases) and control groups (2072 cases) were administered Aidi injection plus GBC and GBC, respectively. The dosage range of Aidi injection was 40–150 mL/time. The treatment time per cycle was 5, 8, 10, 14, 21, 28, or 42 days and treatment cycles were 1–6 cycles through intravenous injection. A total of 49 studies reported tumour responses, including 38 records (WHO guidelines), 8 records (RECIST), and 3 records (not report the guidelines) (Miller et al. 1981; Watanabe et al. 2003). QOL was reported in 24 records that included 1869 cases. In addition, 40 records reported ADRs, including 23 records (WHO guidelines), four records (CTC-AE3.0 criteria), and 13 records (not report the guidelines) (Trotti et al. 2003) (Table 1).

**Table 1.** Characteristics of the included trials.

Study (first author, years)	E/C	Sample size (M/F)	Age (year)	Intervention and control protocol			Reported outcomes (evaluation criteria)
				Experimental	Aidi(D/D/C)	Control	
Chen 2016	30/30	36/24	42–76	Aidi + GP	50 ml × 8 d × 2	GP	ORR (WHO), DCR (WHO)
Chen and Wang 2014	40/40	52/28	41.6 ± 4.72	Aidi + GP	60 ml × 42 d × 1	GP	ORR (WHO), DCR (WHO), QOL
Ding et al. 2011	18/22	27/13	53	Aidi + GP	50 ml × 10 d × 2	GP	ORR (WHO)
Fan et al. 2011	41/38	54/25	E: 39 − 71 C: 41 − 73	Aidi + GP	50 ml × 21 d × 2–4	GP	ORR (WHO), DCR (WHO), QOL
Fang 2016	45/45	NR	E: 40 − 70 C: 43 − 69	Aidi + GP	50 ml × 10 d × 2	GP	ORR (WHO), DCR (WHO), ADRs (CTCAE3.0)
Fu 2012	35/35	NR	70.2 ± 5.6	Aidi + GP	50 ml × 14 d × 2	GP	ORR (WHO), DCR (WHO)
Geng et al. 2020	45/45	61/29	E: 66.95 ± 14.19 C: 66.58 ± 14.26	Aidi + GP	50 ml × 14 d × 4	GP	ORR (NR), DCR (NR), ADRs (NR)
Guo 2020	51/51	58/44	E: 57.52 ± 2.17 C: 58.18 ± 3.11	Aidi + GP	60 ml × 14 d × 4	GP	ADRs (NR)
Han et al. 2015	36/36	39/33	E: 55.4 ± 3.7 C: 54.9 ± 4.2	Aidi + GP	50 ml × 28 d × 3	GP	ORR (WHO), DCR (WHO), ADRs (WHO)
He et al. 2011	29/23	29/23	E: 21 − 73 C: 29 − 74	Aidi + GP	50–100 ml × 15 d × 2–3	GP	ORR (WHO), DCR (WHO), QOL, ADRs (WHO)
Hong et al. 2010	90/70	82/78	38–70	Aidi + GP	60 ml × 14 d × 2	GP	ORR (WHO), DCR (WHO), QOL
Huang et al. 2017	39/40	46/33	E: 58.46 ± 7.43 C: 58.74 ± 7.68	Aidi + GP	60 ml × 21 d × 3	GP	ORR (RECIST), DCR (RECIST), ADRs (WHO)
Jiang et al. 2011	32/30	39/23	E: 68.2 C: 67.6	Aidi + GP	100 ml × 14 d × 2	GP	ORR (NR), DCR (NR)
Ju et al. 2013	34/34	36/32	E: 69.5 ± 8.1 C: 67.4 ± 7.8	Aidi + GP	50 ml × 14 d × 2	GP	ORR (WHO), DCR (WHO), QOL
Lai 2013	70/70	73/67	E: 49 − 79 C: 45 − 75	Aidi + GP	50 ml × 14 d × 2	GP	ORR (WHO), DCR (WHO), QOL, ADRs (WHO)
Li et al. 2016	35/35	43/27	E: 62.15 ± 3.64 C: 64.09 ± 3.14	Aidi + GP	100 ml × 14 d × 2	GP	ORR (NR), DCR (NR), QOL, ADRs (NR)
Li and Yang 2014	27/27	32/22	62.3 ± 4.9	Aidi + GP	50 ml × 8–10 d × 4	GP	ORR (RECIST), DCR (RECIST), ADRs (CTCAE3.0)
Li et al. 2008	53/51	73/31	E: 32 − 79 C: 31 − 77	Aidi + GO	60 ml × 10 d × 2	GO	ORR (WHO), DCR (WHO), QOL, ADRs (NR)
Li et al. 2010	36/36	39/33	E: 29 − 75 C: 32 − 73	Aidi + GP	50–100 ml × 15 d × 2–3	GP	ORR (WHO), DCR (WHO), QOL, ADRs (WHO)
Li et al. 2016	47/47	53/41	E: 55.2 ± 2.4 C: 53.4 ± 2.7	Aidi + GP	50–100 ml × 28 d × 1	GP	ORR (WHO), DCR (WHO), ADRs (WHO)
Liu et al. 2010	32/32	37/27	E: 45 − 73 C: 47 − 75	Aidi + GP	50 ml × 14 d × 4	GP	ORR (WHO), DCR (WHO), ADRs (NR)
Liu and Zhang 2014	24/24	30/18	35–80	Aidi + GP	60 ml × 21 d × 2	GP	QOL (NR)
Liu et al. 2019	44/44	54/34	E: 59.11 ± 6.59 C: 59.07 ± 6.45	Aidi + GP	50 ml × 5 d × 2	GP	ORR (NR), DCR (NR)
Lu et al. 2006	33/29	41/21	E: 29 − 75 C: 31 − 76	Aidi + GP	50–100 ml × 10–15 d × 3	GP	ORR (WHO), DCR (WHO), QOL, ADRs (NR)
Lv et al. 2009	29/29	37/21	E: 65 − 72 C: 60 − 75	Aidi + GP	50 ml × 15 d × 2	GP	ORR (WHO), DCR (WHO), QOL, ADRs (WHO)
Lv et al. 2018	30/30	35/25	E: 67.5 ± 13.6 C: 66.9 ± 15.2	Aidi + GP	50 ml × 14 d × 1	GP	ORR (NR), DCR (NR)
Lv et al. 2009	30/30	42/18	45–70	Aidi + GP	80 ml × 10 d × 2	GP	ORR (WHO), DCR (WHO), QOL, ADRs (WHO)
Ma 2017	42/42	55/29	E: 60.13 ± 8.43 C: 59.49 ± 8.76	Aidi + GP	50 ml × 28 d × 4	GP	ORR (NR), DCR (NR), ADRs (NR)
Ma and Jiang 2016	33/35	39/29	E: 68.9 ± 10.4 C: 66.9 ± 10.6	Aidi + GP	60 ml × 14 d × 1	GP	ORR (WHO), DCR (WHO), ADRs (NR)
Ma et al. 2010	24/24	39/9	E: 58.7 C: 59.1	Aidi + GP	40 ml × 10 d × 2	GP	ORR (WHO), DCR (WHO), ADRs (WHO)
Ning et al. 2015	31/31	49/13	E: 47 − 75 C: 45 − 72	Aidi + GP	50 ml × 14 d × 3	GP	ORR (WHO), DCR (WHO), ADRs (WHO)
Pan et al. 2012	38/42	55/25	E: 39 − 79 C: 42 − 81	Aidi + GP	50 ml × 14 d × 3	GP	ORR (WHO), DCR (WHO), QOL, ADRs (WHO)
Pei 2012	40/40	47/33	E: 57.1 ± 8.3 C: 59.2 ± 6.7	Aidi + GP	50 ml × 8 d × 2	GP	ORR (RECIST), DCR (RECIST)
Shi et al. 2010	28/28	47/9	E: 61.5 C: 60.5	Aidi + GP	50 ml × 14 d × 2	GP	ORR (WHO), DCR (WHO), QOL, ADRs (WHO)
Song et al. 2009	30/30	36/24	53–76	Aidi + GP	50 ml × 14 d × 2	GP	ORR (WHO), DCR (WHO), QOL, ADRs (WHO)
Su and Zhang 2019	41/41	54/28	E: 56.24 ± 9.07 C: 55.08 ± 8.13	Aidi + GP	150 ml × 21 d × 4–6	GP	ORR (RECIST), DCR (RECIST), QOL, ADRs (NR)
Sun et al. 2012	34/34	42/26	E: 60 − 83 C: 62 – 86	Aidi + GP	50 ml × 10 d × 2	GP	ORR (RECIST), DCR (RECIST), QOL, ADRs (CTCAE3.0)
Wang 2012	25/24	35/14	E: 56.8 ± 9.1 C: 57.8 ± 10.2	Aidi + GP	60 ml × 14 d × 3	GP	ORR (WHO), DCR (WHO), ADRs (NR)
Wang and Peng 2012	36/36	46/26	32 − 74	Aidi + GP	80 ml × 10 d × 4	GP	ORR (RECIST), DCR (RECIST), QOL, ADRs (WHO)
Wen 2014	45/45	64/26	E: 67.8 ± 6.4 C: 68.3 ± 5.7	Aidi + GP	50 ml × 21 d × 2	GP	ORR (RECIST), DCR (RECIST), QOL, ADRs (CTCAE3.0)
Wen et al. 2009	38/38	52/24	32–77	Aidi + GP	50 ml × 8–10 d × 2	GP	ORR (WHO), DCR (WHO), QOL, ADRs (WHO)
Wu and Chen 2017	67/68	83/52	E: 59.4 ± 5.5 C: 58.6 ± 5.2	Aidi + GP	100 ml × 10 d × 4	GP	ORR (NR), DCR (NR), ADRs (NR)
Wu et al. 2017	109/109	137/78	E: 54.3 ± 14.2 C: 53.1 ± 13.1	Aidi + GP	50 ml × 14 d × 3	GP	ADRs (NR)
Xiao 2006	39/29	48/20	65–79	Aidi + GP	60–80 ml × 10d × 2	GP	ORR (WHO), DCR (WHO), QOL, ADRs (WHO)
Yang et al. 2008	30/27	39/18	34–82	Aidi + GP	80 ml × 8 d × 2	GP	ORR (WHO), DCR (WHO), QOL
Zhang 2009	32/31	44/19	E: 32 − 79 C: 31 – 71	Aidi + GP	80 ml × 14 d × 2	GP	ORR (WHO), DCR (WHO), ADRs (WHO)
Zhang 2012	41/42	63/20	E: 57.2 ± 9.4 C: 58.2 ± 10.3	Aidi + GP	60 ml × 14 d × 3	GP	ORR (WHO), DCR (WHO), ADRs (NR)
Zhang S 2016	25/25	NR	NR	Aidi + GP	50 ml × 10 d × 4	GP	ORR (RECIST), DCR (RECIST)
Zhang X 2016	19/19	21/17	E: 55.68 ± 4.54 C: 54.62 ± 5.95	Aidi + GP	50 ml × 30 d × 1	GP	ORR (WHO), DCR (WHO), ADRs (NR)
Zhang et al. 2018	40/40	43/37	E: 54.67 ± 3.24 C: 54.71 ± 3.26	Aidi + GP	60 ml × 10 d × 2	GP	ADRs (WHO)
Zhang and Wang 2008	39/35	43/31	E: 21 − 73 C: 29 – 74	Aidi + GP	50–100 ml × 15 d × 2–3	GP	ORR (WHO), DCR (WHO), QOL, ADRs (WHO)
Zhang et al. 2018	52/52	64/40	E: 62.24 ± 2.70 C: 62.19 ± 2.47	Aidi + GP	60–100 ml × 10 d × 4	GP	ORR (NR), DCR (NR), ADRs (NR)
Zhao and Li 2019	43/43	55/31	E: 64.02 ± 2.34 C: 63.26 ± 2.57	Aidi + GP	50 ml × 21 d × 2	GP	ORR (NR), DCR (NR)
Zou et al. 2006	42/39	56/25	E: 37 − 72 C: 35 – 73	Aidi + GP	80 ml × 14 d × 3	GP	ORR (WHO), DCR (WHO), QOL, ADRs (WHO)

Note: NSCLC: non-small cell lung cancer; E/C: experimental group (Aidi injection plus gemcitabine-based chemotherapy)/control group (gemcitabine-based chemotherapy); M/F: male/female; GP: gemcitabine and cisplatin; GO: gemcitabine and oxaliplatin; Aidi(D/D/C): Aidi injection (Dose/Days/ Cycles); WHO: World Health Organisation guidelines for solid tumour responses; RECIST: Response Evaluation Criteria in Solid Tumours; CTCAE: Common terminology criteria for adverse events version; ORR: objective response rate; DCR: disease control rate; QOL: quality of life; ADRs: adverse drug reactions.

### The risk of methodological bias

**Figure 2.**
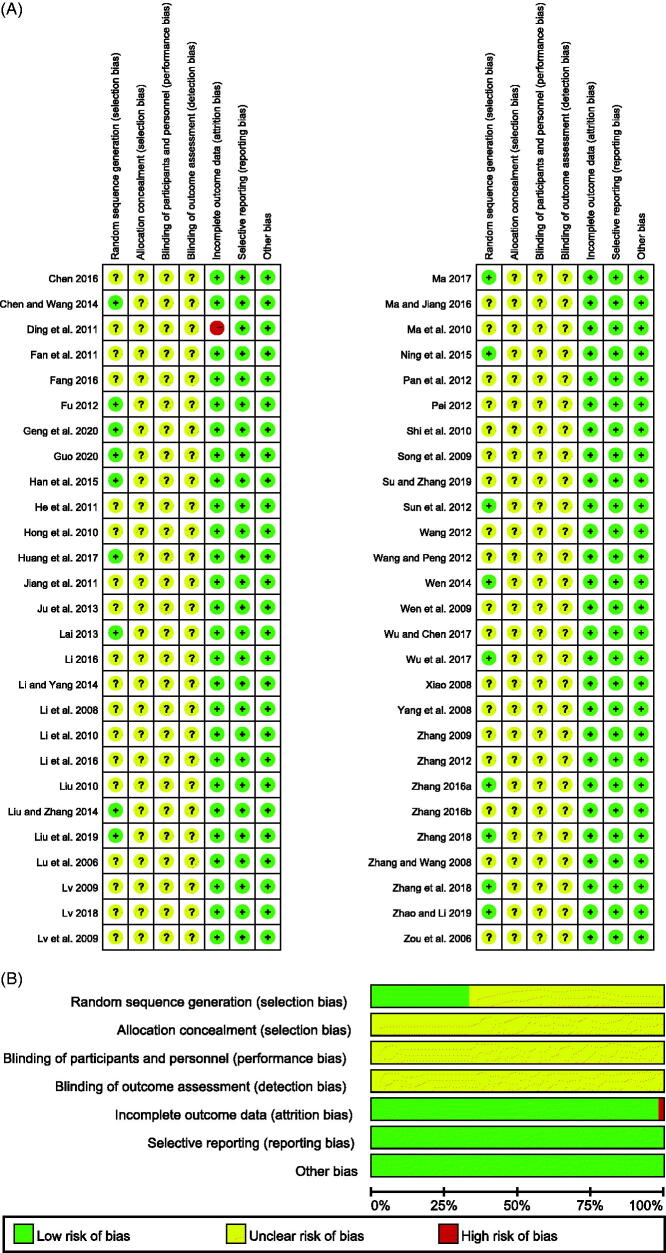
Risk of bias of included studies. (A) Risk of bias summary: judgments about each bias item for each study; (B) Risk of bias summary graph.

As shown in Figure 2, the Cochrane Risk of Bias Assessment Tool was employed to assess the risk of bias and methodologic quality in all 54 RCTs. The results of the assessment indicated that an unclear risk of bias may have existed in some areas. Eighteen studies (Fu 2012; Sun et al. 2012; Lai 2013; Chen and Wang 2014; Liu and Zhang 2014; Wen 2014; Han et al. 2015; Ning et al. 2015; Zhang S 2016; Huang et al. 2017; Ma 2017; Wu and Chen 2017; Zhang 2018; Zhang et al. 2018; Liu et al. 2019; Zhao and Li 2019; Geng et al. 2020; Guo 2020) clearly described the methods of random allocation. Selection bias and Performance bias were unclear in all studies. Attribution bias, reporting bias and other biases were unclear, except one study (Ding et al. 2011) only report ORR which is the index of tumour responses.

### Tumour responses

**Figure 3.**
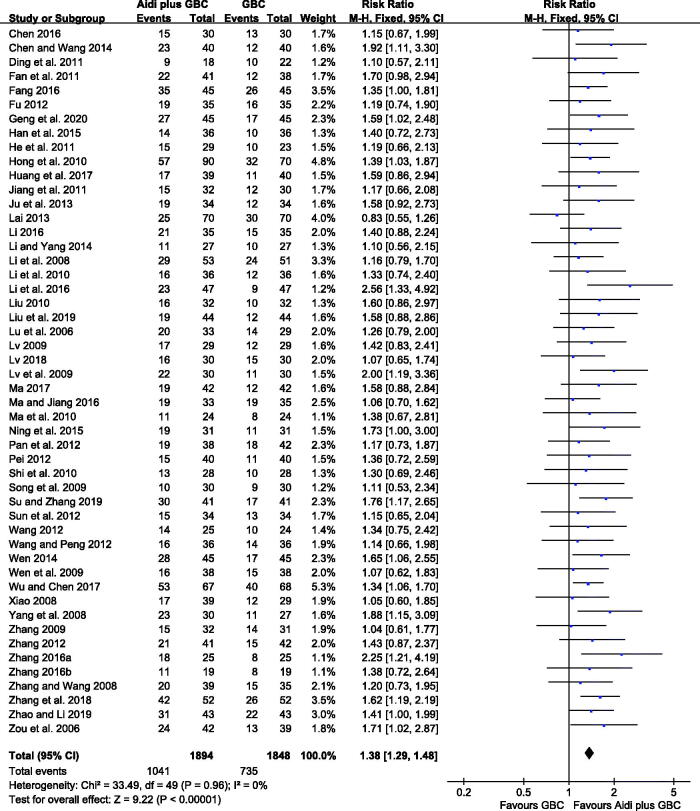
Meta-analysis on the ORR in the Aidi plus GBC vs. GBC.

In all of the included studies, 50 trials that involved 3742 cases accounted for the ORR. According to the results, no statistical heterogeneity was detected among the trials (*I^2^* = 0%). Therefore, the FEM was adopted to calculate the data. Aidi injection plus GBC significantly increased the ORR compared with chemotherapy alone (RR = 1.38, 95% CI 1.29–1.48, *p* < 0.00001, Figure 3).

**Figure 4.**
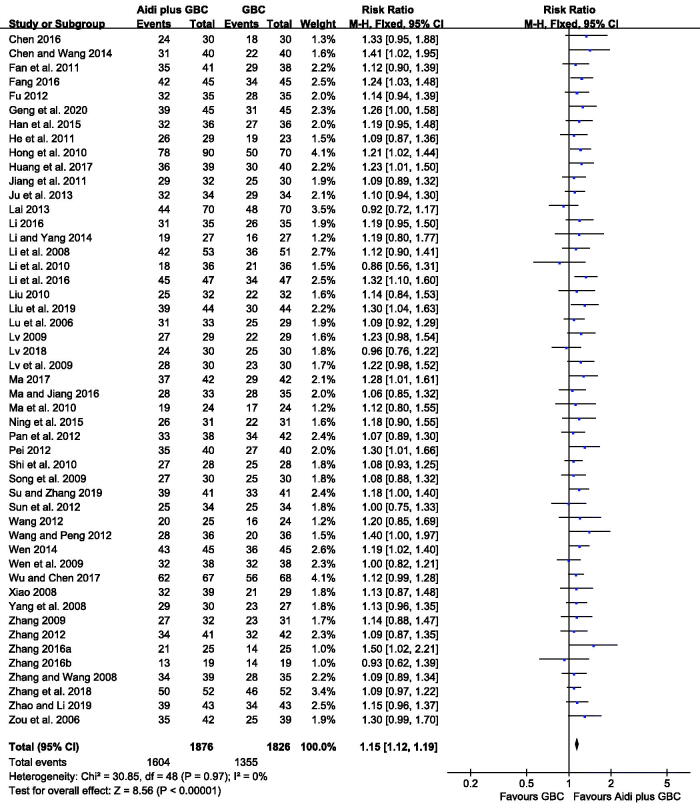
Meta-analysis on the DCR in the Aidi plus GBC vs. GBC.

DCR were reported in 49 studies, with 3702 cases. According to the result, minimal statistical heterogeneity was detected among the trials (*I^2^* = 0%). Therefore, FEM was adopted to calculate the data. Aidi injection plus GBC significantly increased the DCR, compared with chemotherapy alone (RR = 1.15, 95% CI 1.12–1.19, *p* < 0.00001, Figure 4).

### Quality of life (QOL)

**Figure 5.**
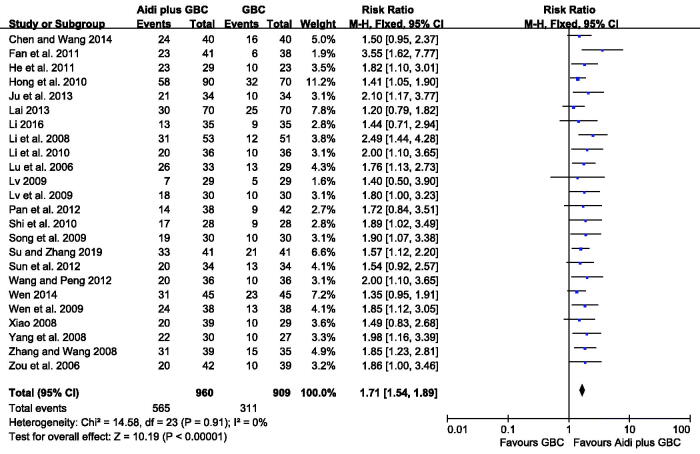
Meta-analysis on the QOL in the Aidi plus GBC vs. GBC.

QOL was reported in 24 studies with 1869 cases, which was assessed in strict accordance with the KPS Scale (Yates et al. 1980). According to the result, minimal statistical heterogeneity was detected among the trials (*I^2^* = 0%). Therefore, FEM was adopted to calculate the data. Aidi injection plus GBC significantly increased the QOL, compared with chemotherapy alone (RR = 2.83, 95% CI 2.33–3.43, *p* < 0.00001, Figure 5).

### Adverse drug reactions (ADRs)

ADRs, including gastrointestinal toxicity, thrombocytopenia, neutropenia, liver or renal injury, and anaemia, were reported in 40 trials (Table 2). There was statistical heterogeneity in gastrointestinal toxicity (*I^2^* = 27%), neutropenia (*I^2^* = 34%) and no heterogeneity in other ADRs (*I^2^* = 0%). Therefore, FEM was adopted to calculate the ADR data. The meta-analysis results indicated that the treatment with Aidi injection plus GBC was associated with a lower the risk of gastrointestinal toxicity (RR = 0.64, 95% CI 0.59–0.70, *p* < 0.0001), thrombocytopenia (RR = 0.49, 95% CI 0.37–0.65, *p* < 0.0001), neutropenia (RR = 0.63, 95% CI 0.58–0.69, *p* < 0.0001), liver injury (RR = 0.48, 95% CI 0.38–0.62, *p* < 0.0001), renal injury (RR = 0.48, 95% CI 0.31–0.74, *p* = 0.0008), and anaemia (RR = 0.49, 95% CI 0.34–0.70, *p* < 0.0001) than GBC alone did. The differences were statistically significant.

**Table 2.** Meta-analysis results of ADRs.

Outcomes	Trials	Aidi plus GBC (Evens/Total)	GBC (Evens/Total)	SM	RR (95% CI)	*I^2^*	*p*
Gastrointestinal toxicity	33	458/1352	703/1332	FEM	0.64 (0.59, 0.70)	27%	<0.00001^*^
Thrombocytopenia	17	134/653	198/647	REM	0.62 (0.46, 0.83)	58%	0.001^*^
Neutropenia	38	480/1535	749/1513	FEM	0.62 (0.57, 0.68)	33%	<0.00001^*^
Liver injury	20	123/869	215/861	FEM	0.57 (0.47 0.69)	0%	<0.00001^*^
Renal injury	7	27/325	56/317	FEM	0.48 (0.31, 0.74)	0%	0.0008^*^
Anaemia	7	61/370	105/369	FEM	0.58 (0.44, 0.76)	0%	0.0001^*^

Note: Aidi: Aidi injection; GBC: gemcitabine-based chemotherapy; RR, relative ratio; REM: random-effects model; FEM: fixed-effects model. *Favours Aidi plus GBC group with statistical significance.

### Subgroup analysis of ORR and DCR

**Figure 6.**
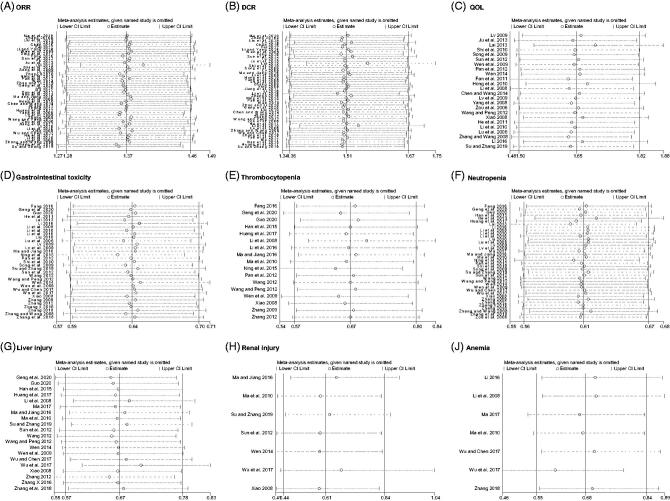
Sensitivity analysis. (A) ORR; (B) DCR; (C) QOL; (D) Gastrointestinal toxicity; (E) Thrombocytopenia; (F) Neutropenia; (G) Liver injury; (H) Renal injury; (I) Anaemia.

In order to prove the clinical heterogeneity and its influence on ORR and DCR the usages (days and doses) of Aidi injection were used as the basis for subgroup analysis. All of the studies were grouped according to the usage days of Aidi injection: 10–30 d, 31–60 d, and > 60 d. Subgroup analysis indicated that the usage of 10–30 d, 31–60 d and > 60 d, of Aidi injection, could increase the ORR and DCR (Table 3). Another group approach was based on the usage doses of Aidi injection: 40, 50, 60, 80, and 50–150 mL. The results showed that only the use of 50, 60, 80, and 50–150 mL of Aidi injection could increase the ORR and DCR (Table 3). The sensitivity analysis did not show the significance for ORR, DCR, and QOL, which showed similar results to those in the subgroup analysis (Figure 6).

**Table 3.** Subgroups analysis of ORR and DCR.

Subgroups	Objective response rate (ORR)						Disease control rate (DCR)					
	Trials	Study event rates		RR (95% CI)	*I* ^2^	*p*	Trials	Study event rates		RR (95% CI)	*I* ^2^	*p*
		Aidi plus GBC	GBC					Aidi plus GBC	GBC			
Totality	50	1041/1894	735/1848	1.38 (1.29, 1.48)	0%	<0.00001	49	1604/1876	1355/1826	1.15 (1.12, 1.19)	0%	<0.00001*
Subgroups analysis via doses												
Aidi injection (40 ml/times)	1	11/24	8/24	1.38 (0.67, 2.81)	NA	0.38	1	19/24	17/24	1.12 (0.80, 1.55)	NA	0.51
Aidi injection (50 ml/times)	26	478/929	359/934	1.34 (1.21, 1.48)	0%	<0.00001	25	772/911	676/912	1.14 (1.09, 1.20)	0%	<0.00001*
Aidi injection (60 ml/times)	7	180/321	123/302	1.36 (1.16, 1.61)	0%	0.0002	7	269/321	214/302	1.18 (1.08, 1.29)	0%	0.0002*
Aidi injection (80 ml/times)	5	100/170	63/163	1.52 (1.20, 1.92)	24%	0.004	5	147/170	114/163	1.23 (1.10, 1.38)	0%	0.0003*
Aidi injection (50–150 ml/times)	11	272/450	182/425	1.42 (1.25, 1.62)	0%	<0.00001	11	397/450	334/425	1.12 (1.06, 1.19)	0%	<0.0001*
Subgroups analysis via treatment time												
10–30 days	27	517/996	389/964	1.28 (1.16, 1.41)	0%	<0.00001	26	838/984	704/948	1.15 (1.10, 1.20)	0%	<0.00001*
31–60 days	18	422/699	284/687	1.46 (1.31, 1.63)	0%	<0.00001	19	622/734	532/719	1.14 (1.09, 1.21)	0%	0.0002*
>60 days	5	102/199	62/197	1.63 (1.28, 2.08)	0%	<0.0001	4	144/158	119/159	1.22 (1.10, 1.35)	0%	<0.00001*

Note: Aidi: Aidi injection; GBC: gemcitabine-based chemotherapy; RR, relative ratio; CI: confidence interval. *Favours Aidi plus GBC group with statistical significance.

**Figure 7.**
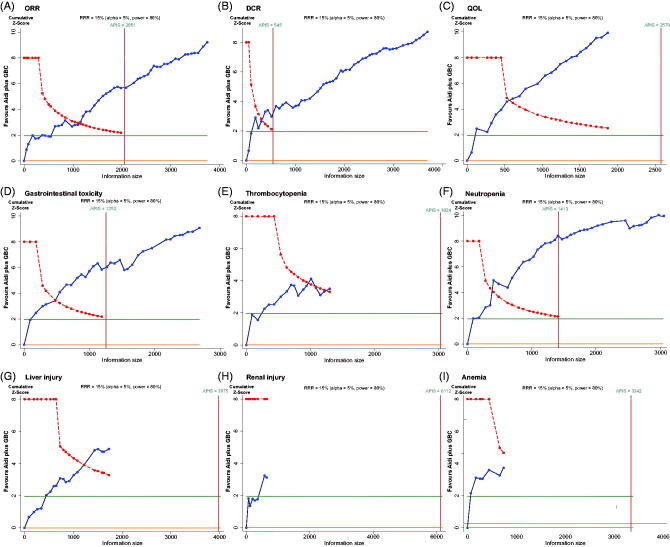
Trial sequential analysis. (A) ORR; (B) DCR; (C) QOL; (D) Gastrointestinal toxicity; (E) Thrombocytopenia; (F) Neutropenia; (G) Liver injury; (H) Renal injury; (I) Anaemia.

This meta-analysis employed TSA boundaries to evaluate the robustness of the results. The required information size (RIS) was calculated. The type I error rate was defined as 5%, the type II error rate was set as 20%, and the relative risk reduction (RRR) was derived from the meta-analysis. As shown in Figure 7(A–C), the *Z*-score curve (blue line) crossed the statistical significance boundary (red polylines), the required information size (vertical red line), and the conventional statistical significance boundary corresponding to a two-sided *p*-value of 0.05 (dark green lines). The results indicated that the improvement of ORR, DCR, and QOL in stage III-IV NSCLC patients with Aidi injection could be considered conclusive with the existing evidence. As shown in Figure 7(D–G), the reduction in gastrointestinal toxicity, thrombocytopenia, neutropenia, and liver injury were definite and well documented. Although the required information size for liver injury and thrombocytopenia did not achieve the expected value, positive results were obtained ahead of time. More research is needed to demonstrate the reduction in renal injury and anaemia (Figure 7(H,I)).

**Figure 8.**
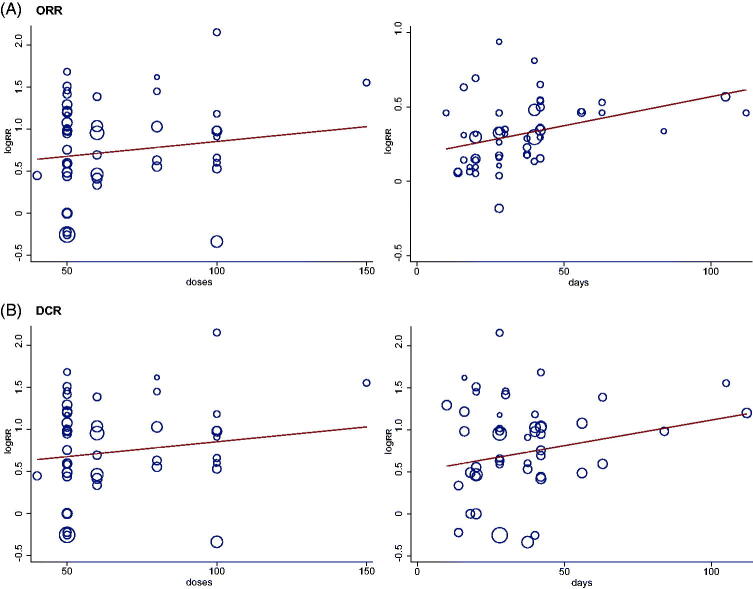
Meta-regression analysis of ORR and DCR.

Increasing the days or doses of Aidi injection could not improve the ORR, DCR and QOL, through the meta-regression analysis (ORR: LogRR = −0.032–0.074 doses, [*u* = 0.021, *p* = 0.434], ORR: LogRR = 0.016–0.235 total days, [*u* = 0.126, *p* = 0.025], DCR: LogRR = −0.107–0.200 doses, [*u* = 0.047, *p* = 0.542], DCR: LogRR = −0.148–0.406 total days, [*u* = 0.129 *p* = 0.355], QOL: LogRR = −0.060–0.109 doses, [*u* = 0.024, *p* = 0.555], QOL: LogRR = −0.159–0.156 total days, [*u* = −0.001, *p* = 0.985]) (Figure 8).

### Publication bias analysis

**Figure 9.**
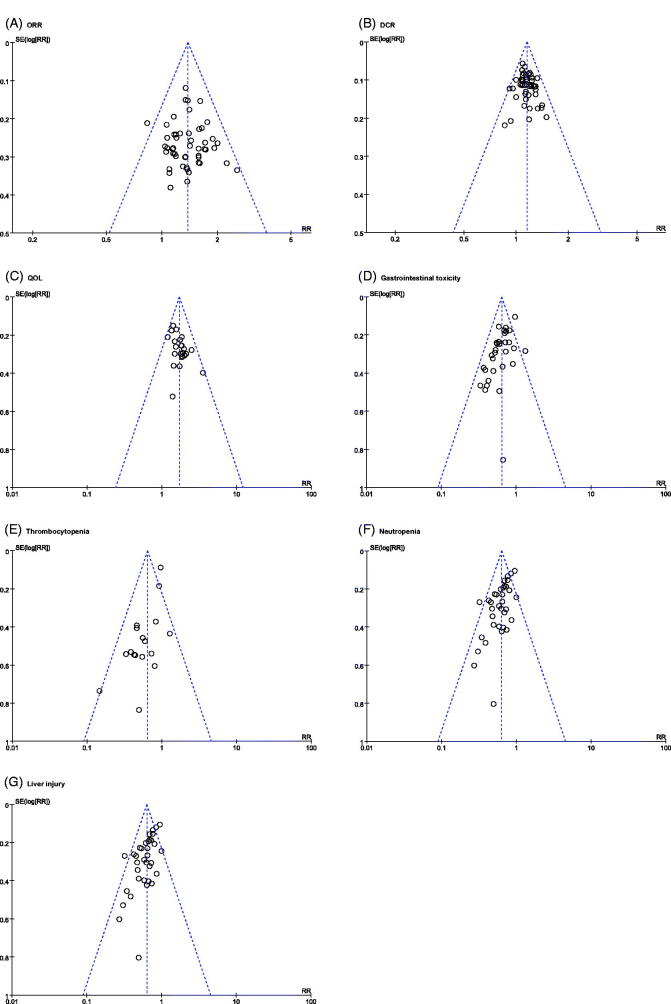
The publication bias analysis.

As displayed in the funnel plots of ORR, DCR, QOL, thrombocytopenia, and liver injury, the symmetrical figures indicated that there was no publication bias (Figure 9(A,B,C,E,G)). The ORR, DCR, QOL, thrombocytopenia, and liver injury were objectively reported. As displayed in the funnel plots of neutropenia and gastrointestinal toxicity, the significantly asymmetrical figures indicated that there had publication bias (Figure 9(D,F)). Furthermore, neutropenia and gastrointestinal toxicity were overestimated.

### Quality of evidence

In all included studies, only 19 studies clearly explained the randomisation method, while others were not mentioned. On account of the sensitivity analysis results indicating good robustness, the level of all outcomes should rate down only one level in the limitation of the study design. Statistical heterogeneity was detected in gastrointestinal toxicity, thrombocytopenia, and neutropenia. Due to the robustness of the results, the level was not rated in the domain of inconsistency. There was publication bias in gastrointestinal toxicity, neutropenia, and liver injury, which were overestimated, and the results had good robustness. Therefore, the evidence was not provided. The total sample size of renal injury and anaemia did not reach the optimal information size, and the sample size for each indicator was less than 300 cases, therefore, the evidence was rated down by one level. None of the outcomes was eligible for upgrade. Overall, expect for renal injury and anaemia, the others’ quality of evidence was moderate (Table 4).

**Table 4.** GRADE evidence profile of clinical efficacy and safety.

Out comes (trials)	Quality assessment					No of patients		Clinical efficacy and safety		Quality
	Risk of bias	Inconsistency	Indirectness	Imprecision	Publication bias	Aidi plus GBC	GBC	Relative ratio (95% CI)	Absolute effects	
ORR (50)	Serious^a^	No serious	No serious	No serious	None	1041/1894 (55.0%)	735/1848 (39.8%)	1.38 (1.29–1.48)	151 more per 1000 (from 115 more to 191 more)	⊕⊕⊕ moderate
DCR (49)	Serious^a^	No serious	No serious	No serious	None	1604/1876 (85.5%)	1355/1826 (74.2%)	1.15 (1.12–1.19)	111 more per 1000 (from 89 more to 141 more)	⊕⊕⊕ moderate
QOL (24)	Serious^a^	No serious	No serious	No serious	None	565/960 (58.9%)	311/909 (34.2%)	1.71 (1.54–1.89)	243 more per 1000 (from 185 more to 304 more)	⊕⊕⊕ moderate
Gastrointestinal toxicity (33)	Serious^a^	No serious^b^	No serious	No serious	None^c^	458/1352 (33.9%)	703/1332 (52.8%)	0.64 (0.59–0.70)	190 fewer per 1000 (from 158 fewer to 216 fewer)	⊕⊕⊕ moderate
Thrombocytopenia (17)	Serious^a^	No serious^b^	No serious	No serious	None	134/653 (20.5%)	198/647 (30.6%)	0.64 (0.54–0.77)	110 fewer per 1000 (from 70 fewer to 141 fewer)	⊕⊕⊕ moderate
Neutropenia (37)	Serious^a^	No serious^b^	No serious	No serious	None^c^	457/1335 (34.2%)	702/1313 (53.5%)	0.63 (0.58–0.69)	198 fewer per 1000 (from 166 fewer to 225 fewer)	⊕⊕⊕ moderate
Liver injury (20)	Serious^a^	No serious	No serious	No serious	None^c^	123/869 (14.3%)	215/861 (25.0%)	0.57 (0.47–0.69)	107 fewer per 1000 (from 77 fewer to 132 fewer)	⊕⊕⊕ moderate
Renal injury (7)	Serious^a^	No serious	No serious	Serious^d^	None	27/325 (8.3%)	56/117 (13.3%)	0.48 (0.31–0.74)	92 fewer per 1000 (from 46 fewer to 122 fewer)	⊕⊕ low
Anaemia (7)	Serious^a^	No serious	No serious	Serious^d^	None	61/370 (16.5%)	105/369 (28.5%)	0.58 (0.44–0.76)	120 fewer per 1000 (from 68 fewer to 159 fewer)	⊕⊕ low

Note: Aidi: Aidi injection; GBC: gemcitabine-based chemotherapy; RR, relative ratio; CI: confidence interval; ORR: objective response rate; DCR: disease control rate; QOL: quality of life.

^a^Most domain trials mentioned applying a randomisation methodology, but few of included study specified the method and none of the trials specified the methods of allocation concealment and the blinding procedures. Therefore, evidence was rated down by only one level.

^b^Considerable heterogeneity and the results had good robustness. Not rated down

^c^Not rated down. The ADRs were over-estimated.

^d^The total sample size did not reach the optimal information size, and the sample size for each indicator was less than 300 cases, and the evidence was rated down by one level.

## Discussion

We have included 54 RCTs involving 4318 stage III-IV NSCLC participants in this meta-analysis. Among the 4318 participants, 2504 were men and 1473 were women aged 21–80 years. The experimental groups (2118 cases) and control groups (2072 cases) were administered Aidi injection plus GBC and GBC. The dosage range of Aidi injection was 40–150 mL/time. The treatment time per cycle was 5, 8, 10, 14, 21, 28, or 42 days, and treatment cycles were 1–6 cycles through intravenous injection. The ORR, DCR, QOL, and ADRs were evaluated after treatment.

GBC is recognised as an important first-line treatment for NSCLC. Can Aidi injection combined with GBC improve tumour responses and QOL in NSCLC patients? The solid tumour responses indicator of ORR and DCR, according to the guidelines, were reported in 50 trials that included 3742 cases and 49 studies with 3702 cases (Miller et al. 1981; Watanabe et al. 2003). QOL was reported in 24 trials with 1869 cases. Aidi injection plus GBC significantly increased the ORR, DCR, and QOL, compared with chemotherapy alone, and minimal statistical heterogeneity was detected. In order to further detect the sources and influences of clinical heterogeneity on ORR, DCR and QOL, subgroup and meta-regression analysis were performed. The subgroup analysis results indicated that Aidi injection, except for the dose of 40 mL treatment, could increase ORR and DCR. More studies are needed to verify the effect of Aidi injection at the dosage of 40 mL each time on NSCLC patients. The meta-regression analysis results indicated that the application of Aidi injection may be one of the sources of clinical heterogeneity, such as the number of treatment days. Sensitivity analysis and TSA did not show the significance for ORR, DCR, and QOL, which showed that the meta-analysis of ORR, DCR, and QOL had good robustness. Furthermore, from the GRADE assessment results, we found that the quality of evidence was moderate.

The meta-analysis results of 40 trials indicated that Aidi injection plus GBC could reduce the risk of gastrointestinal toxicity, neutropenia, liver or renal injury, thrombocytopenia, and anaemia toxicity compared with GBC alone. Sensitivity analysis and TSA results showed that the included studies and sample sizes of gastrointestinal, neutropenia, liver injury and thrombocytopenia toxicity were sufficient. Moreover, almost no statistical heterogeneity was detected. However, further studies are needed to demonstrated the effect of Aidi injection on renal injury and anaemia. The quality of evidence was moderate, except for renal injury and anaemia toxicity.

Compared to previously published studies, the trials and sample sizes were expanded, and the reliability of synthesised results was increased in this meta-analysis (Ma et al. 2009; Wang et al. 2018a). The results showed that Aidi injection plus GBC significantly improved the ORR, DCR, and QOL in NSCLC patients, and significantly reduced the risk of gastrointestinal, neutropenia, liver injury, and thrombocytopenia toxicity.

In China, safety, feasibility, and effectiveness were the prominent characteristic of TCM, after thousands of years of verification. According to the theory of TCM, Aidi mainly has the following three functions: (1) supports vital energy and enhances body resistance; (2) clears away heat and toxic materials; and (3) promotes blood circulation to remove blood stasis. Many studies have shown that cantharidin, ginsenosides, astragalosides, and terpenoid compounds were the main constituents of Aidi. The antitumor and immunomodulatory functions of lung cancer were determined using polysaccharides, extracted from *Acanthopanax senticosus.* (Huang et al. 2011; Zhang et al. 2014; Cichello et al. 2015; Hsia et al. 2016; Xiao et al. 2016; Ge et al. 2017; Meng et al. 2017). Specifically, the following are the pharmacological effects of the active ingredients: (1) the sensitivity of hypoxic lung cancer cells can be increased, cisplatin resistance can be attenuated, and the immune response can be resumed by ginsenoside Rg3; (2) lung cancer cells usually migrate and invade in the body, which can be impaired by *cantharidin*; (3) according to the activation of macrophages and natural killer cells, the *polysaccharides* extracted from *Acanthopanax senticosus* can inhibit tumour metastasis and play an antitumor role; (4) *astragaloside* IV can increase the sensitivity of NSCLC cells to gefitinib; (5) under the action of ginseng, the expression of apoptosis protein is increased and that of anti-apoptotic protein is reduced (Yoon et al. 2004; Hsia et al. 2016; Dai et al. 2017; Jiang et al. 2017; Majeed et al. 2018; Wang et al. 2018b).

There were some limitations in this study: (1) only articles published in Chinese and English were searched; (2) the majority of studies had small sample sizes, ranging from 38 to 218 patients; (3) randomisation, blinding methods, and allocation concealment were not adequately reported in some of the included studies; (4) according to the funnel plot, there is publication bias of some ADRs; this may because a positive result is more likely to be published during the research publication process; and (5) there were so many researchers reporting that the use of TCM plus chemotherapy for NSCLC was common in China. Therefore, there may be some racial bias, as most of the participants in the study were Chinese.

## Conclusions

This study reveals that Aidi injection is a safe, feasible, and effective adjunctive therapy for GBC in stage III-IV NSCLC, improving ORR, DCR, QOL, and alleviating toxicities. Aidi injection as adjunctive therapy for GBC may provide an alternative treatment for patients with stage III-IV NSCLC. Nevertheless, more RCTs with rigorous methods and large sample sizes should be included in order to further assess its effects, due to the intrinsic limitations of the included studies.
